# Integrative Omics Analysis Unravels Microvascular Inflammation-Related Pathways in Kidney Allograft Biopsies

**DOI:** 10.3389/fimmu.2021.738795

**Published:** 2021-11-02

**Authors:** Claire Tinel, Baptiste Lamarthée, Jasper Callemeyn, Elisabet Van Loon, Virginia Sauvaget, Lise Morin, Laïla Aouni, Marion Rabant, Wilfried Gwinner, Pierre Marquet, Maarten Naesens, Dany Anglicheau

**Affiliations:** ^1^ Necker-Enfants Malades Institute, Institut national de la santé et de la recherche médicale (Inserm) U1151, Université de Paris, Paris, France; ^2^ Department of Microbiology, Immunology and Transplantation, Nephrology and Kidney Transplantation Research Group, Katholieke Universiteit (KU) Leuven, Leuven, Belgium; ^3^ Department of Nephrology and Kidney Transplantation, University Hospitals Leuven, Leuven, Belgium; ^4^ Department of Nephrology and Kidney Transplantation, Necker Hospital, Assistance Publique-Hôpitaux de Paris, Paris, France; ^5^ Department of Pathology, Necker Hospital, Assistance Publique-Hôpitaux de Paris, Paris, France; ^6^ Department of Nephrology, Hannover Medical School, Hannover, Germany; ^7^ Institut national de la santé et de la recherche médicale (Inserm), University of Limoges, Limoges University Hospital, Pharmacology & Transplantation, Limoges, France

**Keywords:** kidney transplantation, microRNA, multi-omics, microvascular inflammation, antibody-mediated rejection

## Abstract

In solid-organ transplantation, microRNAs (miRNAs) have emerged as key players in the regulation of allograft cells function in response to injury. To gain insight into the role of miRNAs in antibody-mediated rejection, a rejection phenotype histologically defined by microvascular inflammation, kidney allograft biopsies were subjected to miRNA but also messenger RNA (mRNA) profiling. Using a unique multistep selection process specific to the BIOMARGIN study (discovery cohort, N=86; selection cohort, N=99; validation cohort, N=298), six differentially expressed miRNAs were consistently identified: miR-139-5p (down) and miR-142-3p/150-5p/155-5p/222-3p/223-3p (up). Their expression level gradually correlated with microvascular inflammation intensity. The cell specificity of miRNAs target genes was investigated by integrating their *in vivo* mRNA targets with single-cell RNA sequencing from an independent allograft biopsy cohort. Endothelial-derived miR-139-5p expression correlated negatively with MHC-related genes expression. Conversely, epithelial-derived miR-222-3p overexpression was strongly associated with degraded renal electrolyte homeostasis and repressed immune-related pathways. In immune cells, miR-150-5p regulated NF-κB activation in T lymphocytes whereas miR-155-5p regulated mRNA splicing in antigen-presenting cells. Altogether, integrated omics enabled us to unravel new pathways involved in microvascular inflammation and suggests that metabolism modifications in tubular epithelial cells occur as a consequence of antibody-mediated rejection, beyond the nearby endothelial compartment.

## Introduction

In kidney transplantation (KT), alloimmune injury is usually dichotomized into two types of allograft rejection according to spatial distribution of immune infiltration and information on the presence or absence of donor-specific anti-HLA antibodies. The diagnosis relies on the allograft biopsy with grading of histological lesions according to the Banff International Consensus classification (1). Tubulitis (“t” lesion) and interstitial inflammation (“i” lesion) are hallmark histological features of T cell-mediated rejection (TCMR) and are related to direct cytotoxicity to the tubulointerstitial compartment. Antibody-mediated rejection (ABMR) is most often associated with circulating anti-HLA antibodies with inflammation targeting the microvascular compartment. Active ABMR histological lesions include the presence of mononuclear cells in glomerular capillaries (“g” lesion) and in peritubular capillaries (“ptc” lesion), combinedly referred to as “microvascular inflammation” (MVI). In chronic active ABMR, chronic tissue injury such as transplant glomerulopathy or arterial intimal fibrosis add up to these acute lesions (1). Using the current T-cell targeted immunosuppressive agents, TCMR is considered to have a limited prognostic impact, while ABMR is a major cause of graft failure, which relates to the lack of efficacious therapeutic approaches (2).

In order to find better therapies for MVI and ABMR, better insight in the biological mechanisms underlying these injury processes is needed. High-throughput omics technologies seem to be fit for the purpose, since they allow exact and simultaneous screening of thousands of genes, proteins and metabolites (3). Whole-transcriptome interrogation of kidney allograft biopsies has demonstrated profound mRNA gene expression changes in the injured resident cells or in infiltrating cell populations during ABMR (4). Meanwhile, microRNAs (miRNAs) have emerged as key players in cell metabolism, by regulating messenger RNA (mRNA) at the posttranscriptional level. In fact, miRNAs revolutionized our understanding of the finetuning of the body’s physiological processes involved in proliferation, apoptosis, differentiation and development (5). Not surprisingly, dysregulated miRNA expression results in the development of many pathologies including renal disease (6). As miRNA expression profiles differ between diseased and healthy states, many studies have analyzed miRNAs either alone or in combination with other more traditional markers, not only to diagnose disease and prognosticate disease progression, but also to design potential therapeutic applications (7).

In the context of solid-organ transplantation, miRNAs may be involved in rejection through their role in the regulation of immune cell function and in the response of allograft resident cells to injury (6, 8). Several studies have investigated allograft miRNAs as biomarkers and/or effectors in the settings of TCMR (8, 9), but none have so far focused on MVI and ABMR. In this study, we aimed to integrate different omics levels from kidney allograft biopsies, to gain insight into the role of miRNAs in this deleterious rejection phenotype.

## Materials and Methods

### Study Design

**Figure 1:**
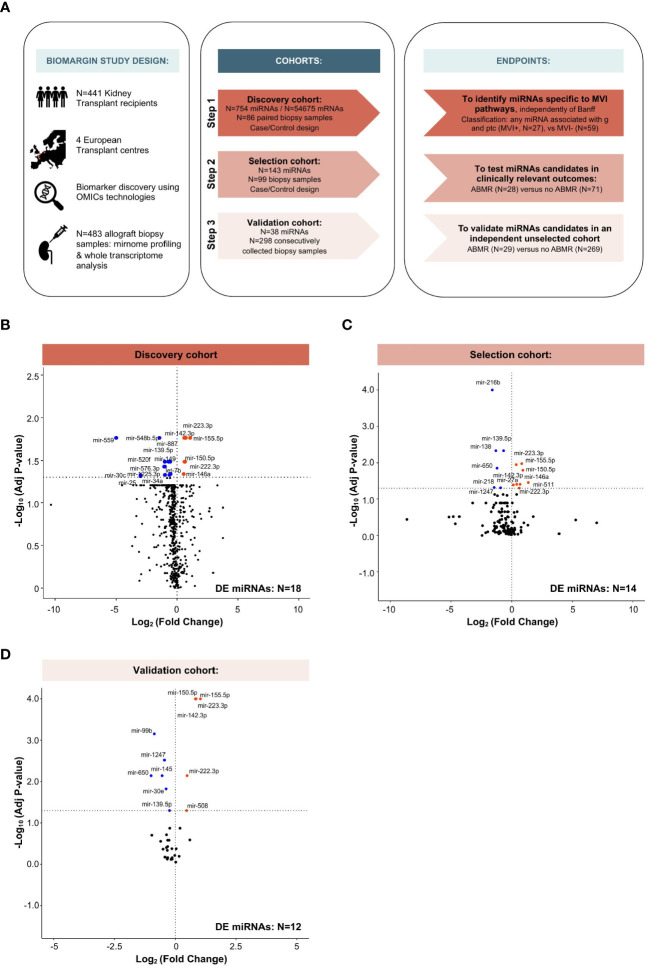
Study design and multi-step identification of MVI-associated miRNAs in kidney allograft tissue. **(A)** Study design, cohorts and specific endpoints. **(B-D)** Volcano Plots displaying the miRNAs differential expression (DE) analysis between MVI+ and MVI- groups **(B)** or ABMR and no ABMR groups **(C, D)** across the indicated cohorts. P-values from a Wilcoxon test, with adjustment for multiple testing using Benjamini & Hochberg false discovery rate (FDR) method. Adjusted P-values < 0.05 were considered as significant (horizontal dashed line).

This study is part of the FP7-funded BIOMArkers of Renal Graft INjuries research program (BIOMARGIN, https://cordis.europa.eu/project/id/305499, ClinicalTrials.gov, number NCT02832661), led by a European research consortium in search of biomarkers of allograft immune lesions in kidney transplant recipients, with diagnostic and/or prognostic value. BIOMARGIN is a multicenter, prospective, multiphase study, performed at four kidney transplant centers in Europe (Necker Hospital, Paris, France; University Hospitals, Leuven, Belgium; Hannover Medical School, Hannover, Germany; and University Hospital, Limoges, France). Samples were prospectively collected at the time of screening and indication biopsies between April 2013 and June 2015 (**Figure 1A**). All transplantations were performed with negative complement-dependent cytotoxicity crossmatches. In these four clinical centers, protocol biopsies were performed at 3, 12, and sometimes at 24 months after transplantation, according to local center practice, in addition to the indication biopsies. The institutional review board at each site approved the study, and all the patients provided written informed consent.

### Study Cohorts

The study was divided into three phases (**Figure 1A**). In the first case-control discovery phase, N=88 biopsy samples were used for global miRNA expression analysis (N=754 miRNAs, excluding potential normalizing miRNAs). We selected samples based on availability and histological criteria (excluding cases with a diagnosis of glomerulonephritis or polyomavirus-associated nephropathy and cases with an unclear diagnosis). Based on local biopsy readings, a first selection was made, which was further refined through central reading by a group of pathologists, independent from the original center. Statistical analysis (see **miRNA selection**) was used to select the extended list of miRNAs, most differentially expressed between histological phenotypes, that were subsequently quantified in the 2nd phase.

A similar case-control study design was used for the second (selection) phase, where targeted miRNA expression analysis (N=143) was performed on N=99 biopsy samples. The same statistical pipeline was applied to select an even more restricted list of miRNAs, to be subsequently quantified in the 3rd phase.

Finally, in the third (validation) cohort, all samples prospectively and consecutively collected according to the BIOMARGIN protocol between June 24, 2014 and July 2, 2015 and with adequate kidney allograft biopsy quality, were assessed for the 38 miRNAs selected as a result of the second selection phase. In this real-life setting cohort, no selection was made based on histology, demographics, time or any factor other than sample availability and quality control criteria (adequacy of biopsy and RNA yield).

### miRNA Selection

A robust statistical pipeline was specifically developed for the BIOMARGIN study in R (R Core Team (10), version 1.0.44), as an extension of the biosigner R package (11). Briefly, in the discovery phase, we carried out a feature selection strategy including univariate and multivariate approaches. For univariate selection, we used a nonparametric test (Wilcoxon-Mann-Whitney test) and a parametric test (Student’s t-test) and computed the results into an “univariate score”. To rank the variables, we considered their false discovery rate (FDR)-adjusted P-value with a significance cutoff of 0.05. For multivariate selection, we carried out five approaches comprising sparse partial least squares (SPLS) (12), support vector machine-recursive feature elimination (SVM-RFE) (13), random forest-recursive feature elimination (RF-RFE) (14), elastic net and shrunken centroids (15). Principal component analysis was used to identify statistical anomalies, resulting in the exclusion of two outliers in the Discovery cohort (BIOS-03-0023, BIOS-06-0238). The prediction scores of each miRNA transcript in each multivariate model were integrated to yield a “multivariate score”. By combining the results of these univariate and multivariate analyses, we were able to rank and select a subset of miRNA candidates to be part of the extended list to be quantified during the next phase.

For this *post-hoc* analysis focusing on MVI pathways, the median normalized expression of each miRNA was compared between cases and controls using the Wilcoxon test in all three cohorts. We controlled for multiple testing using the Benjamini & Hochberg false discovery rate (FDR) method.

### Clinicopathologic Assessment

The individual histological lesion scores were semi-quantitatively assessed according to the Banff 2019 criteria (16). The term “microvascular inflammation” (MVI) was used to refer to any degree of combination of glomerulitis (g) and/or peritubular capillaritis (ptc). All ABMR cases met the first 2 criteria of the Banff 2015 or Banff 2017 classification (histologic evidence of acute tissue injury and evidence of current/recent antibody interaction with vascular endothelium), but not all met the third criterion [serologic evidence of donor-specific antibodies (DSA) and/or C4d staining]. DSAs after transplantation were determined per local center practice, with DSA positivity defined as detectable donor-specific serum anti-HLA antibodies with a mean fluorescence intensity (MFI) value of 500 at the time of biopsy or any time before.

### RNA Extraction From Biopsy Samples for mRNA and miRNA Profiling

Two needle cores were taken at each kidney allograft biopsy. One was used for conventional histological grading and at least half of the other was immediately stored in Allprotect Tissue Reagent (Qiagen Benelux BV, Venlo, The Netherlands). The Allprotect tubes were stored at 4°C (minimum 24 hours to a maximum of 72 hours), and then stored at -20°C until RNA extraction. Total RNA was isolated from the kidney allograft biopsy specimens using the Allprep DNA/RNA/miRNA Universal Kit (Qiagen Benelux BV) on a QIAcube instrument (Qiagen Benelux BV). The quantity (absorbance at 260 nm) and purity (ratio of the absorbance at 230, 260, and 280 nm) of the isolated RNA were measured using the NanoDrop ND-1000 spectrophotometer (Thermo Fisher Scientific/Life Technologies Europe BV, Ghent, Belgium). RNA integrity number (RIN) was evaluated with the Eukaryote nano/pico RNA Kit (Agilent Technologies Belgium NV, Diegem, Belgium) on the Bioanalyzer 2100 instrument (Agilent Technologies BelgiumNV). Quality control indices were not significantly different between the MVI and the No MVI groups [RIN (mean ± SD): 5.6 ± 1.96 *vs* 5.4 ± 1.97, P=0.66; 260/280 ratio (mean ± SD): 1.87 ± 0.06 *vs* 1.87 ± 0.11, P=0.40]. For RNA samples with a low RIN (<6.5), we excluded samples with a 260/280 ratio <1.6. The extracted RNA was subsequently split and stored at -80°C. Half of the RNA extract was used for miRNA profiling and the other half for mRNA transcriptomic analysis. The associated data are available from the Gene Expression Omnibus (GEO, GSE179772, https://www.ncbi.nlm.nih.gov/geo).

### miRNA Profiling

Specific reverse transcription of 150 ng of total RNA was performed using Megaplex™ RT Primers Human Pool A v2.1 (Step 1) or Custom RT Primers Human Pool (Step 2 and 3) (Thermo Fisher Scientific, Les Ulis, France), on a Veriti Thermal Cycler (Applied Biosystems™, Thermo Fisher Scientific). No complementary (c)DNA preamplification was required. miRNA expression was assessed by qPCR, using TaqMan^®^ Array Cards (Applied Biosystems™, Thermo Fisher Scientific), where the primers and probe of each miRNA were spotted and dried in duplicate wells of a microfluidic card. We used TaqMan^®^ Array Human MicroRNA A+B Card Sets v3.0 for the discovery cohort (a two-card set enabling quantitation of 754 unselected human miRNAs) and Custom TaqMan^®^ Array MicroRNA Cards for the selection and validation cohorts. Data analysis was performed by using Expression Suite software version 1.0.3. Assays with a cycle threshold (CT) values >35 were considered to be not expressed. *RNU44 and RNU48* showed consistent expression in all the samples [mean (RNU44+RNU48) coefficient of variation was 3.73% in Array Card A and 3.79% in Array Card B] and were used as housekeeping genes for data normalization across all sub-studies.

### mRNA Transcriptomic Analysis

Transcriptomic analysis was performed by microarray as already described (17). Briefly, total RNA extracted from the biopsy samples was first amplified and biotinylated to complementary RNA (cRNA) using the GeneChip 39 IVT PLUS Reagent Kit (Affymetrix) and hybridized to the Affymetrix GeneChip Human Genome U133 Plus 2.0 Arrays (Affymetrix), which comprised 54,675 probe sets covering the whole genome. We used a Robust Multichip Average (RMA) normalization. The median normalized expression of each mRNA was compared between cases and controls using the Wilcoxon test. We controlled the inflation of risk alpha due to multiple testing by applying the Benjamini & Hochberg false discovery rate (FDR) method. The microarray data were handled in accordance with the Minimum Information About a Microarray Experiment guidelines.

### *In Silico* Analyses

Biological Interpretation Of Multiomics EXperiments (BIOMEX) software was used for transcriptomic data differential analysis, using Ensembl ID annotation (18). Ingenuity Pathway Analysis (IPA, Build: 478438M Content version: 44691306, Qiagen) and OMICSnet (19) were used to determine the sets of genes regulated by the selected miRNAs. OMICSnet was used to perform Gene Ontology analysis with the Reactome pathway database, using the complete pathway (20). Gene networks building was fully unsupervised and relied on the number of reported molecular interactions of each single gene with all other genes in the network. Genes presenting the highest number of interactions were automatically positioned at the center of the network, while the genes with a lower number of interactions were spread into the periphery. The cellular origin of selected miRNAs was investigated using the Fantom5 project (21), which provides a cap analysis of gene expression in human cells and tissues. For miRNAs expression analysis, we considered all kidney-related structural cells and all circulating hematopoietic cells, with no further selection.

### Single-Cell RNA Sequencing Data Analysis

Previously published human single-cell RNA sequencing (scRNA-seq) data from two ABMR biopsies and four healthy references corresponding to transplant surveillance biopsies were used. The associated raw counts or matrices were downloaded respectively from the GEO (GSE145927, https://www.ncbi.nlm.nih.gov/geo) (22) and Kidney Precision Medicine Project (KPMP, https://atlas.kpmp.org/repository). Raw gene expression matrices generated per sample were merged and analyzed using Seurat V4 package (23). The parameters applied to create and filter Seurat objects were as follows: inclusion of cells expressing between 500 and 10000 detected genes and less than 25% mitochondrial transcripts. After filtering, all objects were integrated using the SCTransform integration workflow on Seurat (24). Briefly, 3000 features were used for integration using PrepSCTIntegration function, and the FindIntegrationAnchors function was used to limit batch effect. A full Uniform Manifold Approximation and Projection (UMAP) data set was generated using the DimPlot function and the top 16 principal components. Clusters were built using the FindNeighbors and FindClusters functions with a 0.6 resolution. Cluster identification was performed using the FeaturePlot function by evaluating the expression of specific markers in each cluster. Dot plots were generated using the DotPlot and FeaturePlot plotting functions, with normalized counts in the RNA assay as input data.

### Statistical Analysis

Patient and donor characteristics were described by numbers, percentages and frequencies for categorical variables. We reported continuous variables using means and standard deviations (SD) if normally distributed, medians and interquartile ranges (IQR) for variables with a skewed distribution. We compared their characteristics using the Fisher or the Wilcoxon test when appropriate.

We used the Kruskal-Wallis test to compare the median miRNA expression according to MVI intensity, and the Spearman test for correlation analysis. We considered statistical significance for P-values less than 0.05. We used GraphPad Prism (version 8; GraphPad Software, San Diego, CA) and RStudio (version 4.0.3) for statistical analysis and data presentation. The following R packages were used: heatmap3 (heatmap3_1.1.9) for heatmap analyses, fmsb (fmsb_0.7.0) and scales (scales_1.1.1) packages for radar plots, ggplot2 (ggplot2_3.3.5) for bar graphs, and corrplot (corrplot_0.90) for correlation matrix analysis.

## Results

### Multistep Identification of MVI-Associated miRNAs

Between April 2013 and July 2015, 646 patients enrolled in the BIOMARGIN study provided 716 indication or surveillance biopsies. Inadequate biopsies (N=49), biopsies with no paired sample for research use (N=135) or samples not passing molecular biology QC (N=49) were excluded, leaving 483 samples from 441 individuals available for this study (**Figure 1A**). Baseline and clinical characteristics of the study groups are provided in **Supplementary Tables S1-3**, and the histological characteristics of the biopsies are provided in **Supplementary Tables S4-6**.

In the discovery cohort, a total of 27 patients met the MVI composite endpoint (MVI+), defined as clinical ABMR diagnosis and/or any level of MVI (g and/or ptc >0). The other 59 patients (MVI-) presented various diagnoses comprising T-cell mediated rejection (TCMR, N=8), interstitial fibrosis and tubular atrophy (IFTA, N=20), and normal biopsies (N=31), but no MVI. From the 754 miRNA candidates, 18 miRNAs were differently expressed between MVI+ and MVI- groups (**Figure 1B**). In the selection cohort, a restricted list of 143 miRNAs were quantified in 99 samples. In this selection cohort, 14 miRNAs were differently expressed between cases with (N=28) and without (N=71) ABMR (**Figure 1C**). Finally, in the largest trans-sectional cohort, 38 miRNAs were quantified in 298 samples, of which 12 miRNAs were differentially expressed between ABMR (N=29) *vs* no ABMR (N=269) biopsies (**Figure 1D**).

### Consistent Identification of 6 MVI-Associated MiRNAs and Correlation to Histology

**Figure 2:**
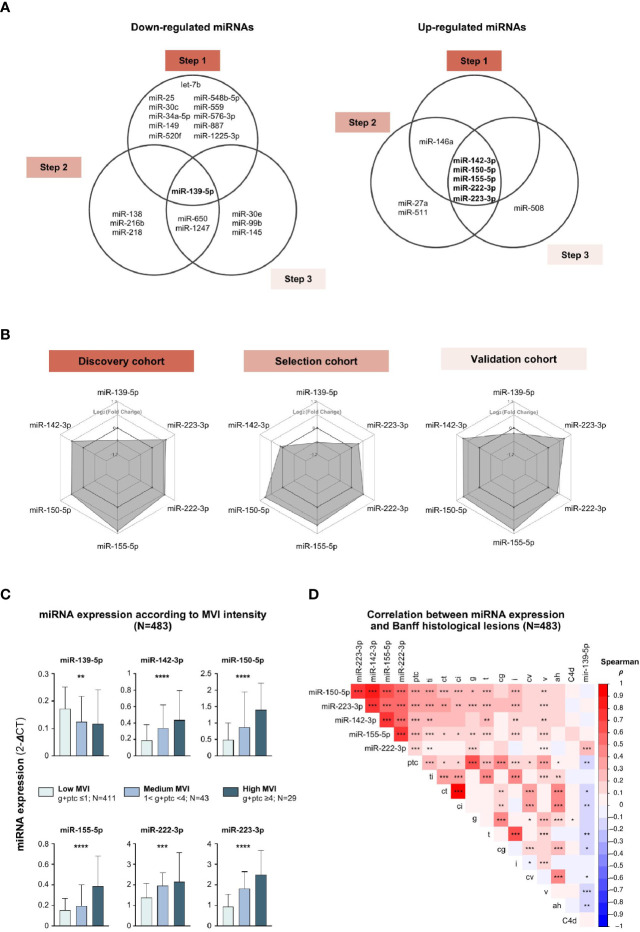
MVI-associated miRNAs profiles correlate with histological lesions. **(A)** Venn diagrams depicting the concordant down-regulated (left) or up-regulated (right) MVI-associated miRNAs across independent cohorts. **(B)** Radar plots showing similar expression profiles of the 6 miRNAs across the 3 cohorts. Each hexagonal line corresponds to a specific differential expression between cases and controls, given as Log_2_ (Fold Change). **(C)** Bar plots showing the expression of individual miRNAs according to MVI intensity with corresponding number of samples. P-values from a Kruskal-Wallis test, *P < 0.05, **P < 0.01, ***P < 0.001. **(D)** Correlogram of the 6 miRNAs expression and Banff histological lesions using Spearman correlation (**P < 0.01, ***P < 0.001, ****P < 0.0001). Colors indicate correlation coefficient (ρ).ah, arteriolar hyalinosis; cg, chronic glomerulopathy; ci, interstitial fibrosis; ct, tubular atrophy; cv, chronic vasculopathy; i, interstitial inflammation; ptc, peritubular capillaritis; ti, total inflammation; v, vasculitis.

Next, we intersected the 3 lists of differentially expressed miRNAs, and identified 6 miRNAs which were consistently associated with MVI or ABMR across the 3 independent cohorts (**Figure 2A**). miR-142-3p, miR-150-5p, miR-155-5p, miR-222-3p and miR-223-3p were overexpressed in MVI/ABMR, while miR-139-5p expression was decreased. Radar plots (**Figure 2B**) display for each step the differential expression between case and controls of the 6 miRNAs and support the robustness of the miRNA profiles across the three independent cohorts.

Next, we studied how the 6 miRNAs’ expression is associated to individual histological lesions across all 483 allograft biopsies taken together. The expression of the 5 up-regulated miRNAs gradually increased with MVI lesions intensity, while the expression of miR-139-5p was negatively correlated (**Figure 2C**). MiR-139-5p/142-3p/150-5p/155-5p/223-3p were not only highly associated with ABMR-related features (g, ptc, C4d deposition), but also with TCMR-related lesions (i, t) and IFTA lesions (ct, ci), suggesting their involvement in common, non ABMR-specific, inflammatory and scarring processes. Alternately, some intrinsic collinearity might by nature exist between the lesions, resulting in our inability to identify true specificity for one lesion. MiR-222-3p did not correlate with histological lesions of TCMR or IFTA (**Figure 2D**).

### Multi-Omics Integration: mRNA-miRNA Interplay in MVI

**Figure 3:**
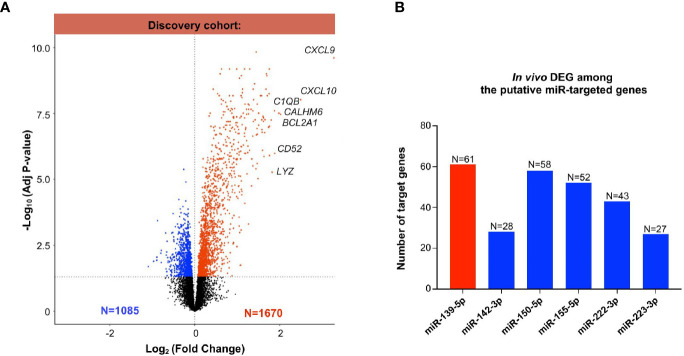
Whole transcriptomic analysis from identical allograft biopsy samples: mRNA/miRNA integration. **(A)** Volcano Plot showing the differentially expressed (DEG) analysis between MVI+ and MVI- samples in the discovery cohort. Blue dots: decreased expression in MVI+. Red dots: increased expression in MVI+. Black dots: non-significant differential expression. **(B)** Integration of miRNAs and mRNAs revealed *in vivo* validated target genes of each individual miRNAs. *In silico* putative target genes of miR-139-5p were intersected with MVI-upregulated genes (red color) whereas putative targets genes of miR-142-3p, 150-5p, 155-5p, 222-3p and 223-3p were intersected with MVI-downregulated genes (blue color). The number of target genes of each list is indicated.

Next, we evaluated the differentially expressed genes between MVI+ and MVI- cases, in the previously published discovery set (25). Of the 54,675 probes, a total of 2755 differentially expressed genes (DEGs) were identified between the MVI+ and MVI- groups, comprising 1085 down-regulated and 1670 up-regulated genes (**Figure 3A**). The most differentially expressed mRNAs in MVI+ samples were *CXCL9* and *CXCL10*, in line with previous reports (17, 26) that these genes are the most overexpressed in ABMR. Next, we used IPA and OMICSnet to identify *in silico* the putative target genes for each miRNA. A total of 4504 mRNAs were predicted to be targeted by at least one of the 6 miRNAs. Then, we integrated these 4504 predicted targets with the DEGs in allograft biopsies. Integrated miRNA/mRNA analysis identified 61 DEGs targeted by miR-139-5p that were significantly increased in MVI+ biopsies. It also identified 28, 58, 52, 43 and 27 DEGs significantly decreased in MVI+ biopsies that were respectively targeted by miR-142-3p, miR-150-5p, miR-155-5p, miR-222-3p, and miR-223-3p (**Figure 3B**).

### Cellular Origin of the MVI-Associated miRNAs

**Figure 4:**
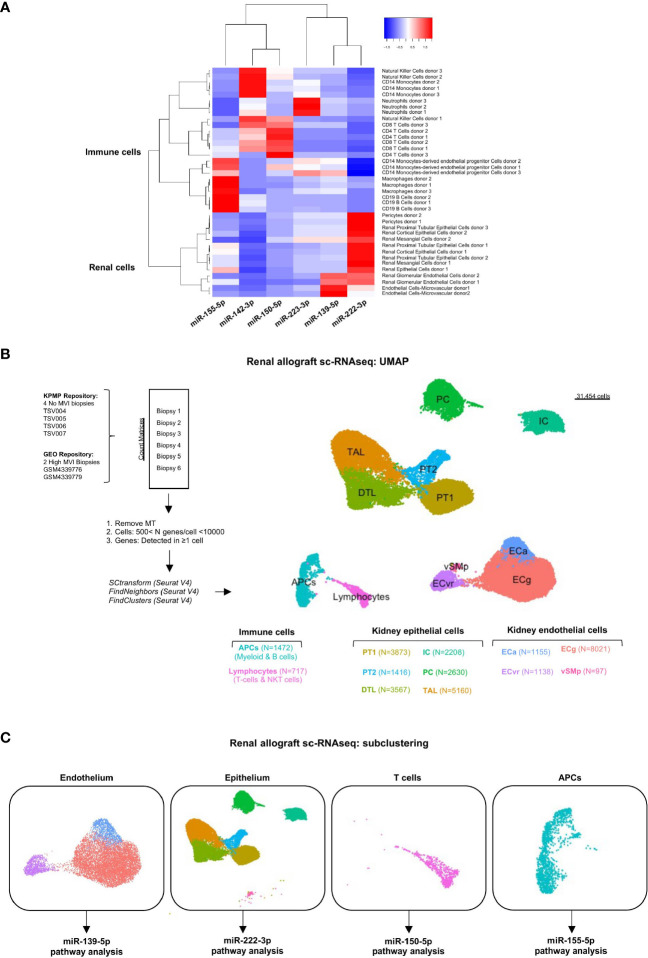
Cellular origins of each individual MVI-associated miRNAs. **(A)** Heatmap analysis of miRNAs expression across tissues using the Fantom5 project (21). Reordering of dendrograms based on hierarchical clustering. Two distinct clusters, immune-derived and renal-derived miRNAs can be distinguished. **(B)** UMAP dimensionality reduction of the different cell type clusters from sc-RNAseq analysis of indicated samples: two High MVI biopsies and four No MVI biopsies. The number of cells in each cluster is provided. **(C)** UMAPs of the four different subclusters corresponding to endothelium, epithelium, T cells and APCs. APCs, antigen-presenting cells; DTL, descending thick limb cells; ECa, activated endothelial cells; ECg, glomerular endothelial cells; ECvr, vasa recta endothelial cells; IC, intercalated cells; PC, principal cells; PT, proximal tubular cells; TAL, thick ascending limb cells; UMAP, Uniform manifold approximation and projection; vSMp, vascular smooth muscle and pericytes.

The cellular origin of the six miRNAs was characterized in various renal cell and hematopoietic cell subtypes using an miRNA atlas available online [Fantom database (27)]. Unsupervised clustering discriminated renal cell-derived miRNAs from immune cell-derived miRNAs (**Figure 4A**). It also revealed one or two dominant cell types for each miRNA. MiR-139-5p appeared to be mainly expressed by glomerular endothelial cells (EC). MiR-222-3p, although its expression was not correlated with tubular inflammation (Banff lesions “t”, **Figure 2D**), was primarily related to renal epithelial cells. The four remaining miRNAs were primarily expressed in peripheral blood mononuclear cells (PBMC) or granulocytes, with miR-142-3p enriched in NK cells and monocytes, miR-150-5p in CD4+ and CD8+ T cells, miR-155-5p in CD19+ B cells and macrophages, and miR-223-3p in neutrophils.

### Single-Cell RNA Sequencing Identified All Renal Cells and Infiltrating Immune Cells

To characterize the potential role of each MVI-associated miRNAs, we analyzed publicly available sc-RNAseq data from two renal allograft biopsies with ABMR and high MVI scores (g2, ptc3) (22). These datasets were integrated with 4 healthy reference biopsies with no MVI. After quality control and filtering, 31545 cells were detected and unsupervised clustering revealed 12 clusters (**Figure 4B**). We used well established markers (28) to identify all the main resident and infiltrating cell subtypes and label them (**Supplementary Figure S2**). Next, we focused on the cell populations from which our six miRNAs were thought to originate from. In this aim, we stratified the cells into 4 major clusters corresponding to endothelium, epithelium, lymphocytes and APCs (**Figure 4C**). In line with previous analysis of these data (22), neither neutrophilic nor NK cell populations were detected in the sc-RNASeq datasets.

### miR-139-5p Controls EC Activation and Antigen-Presentation Pathway During MVI

**Figure 5:**
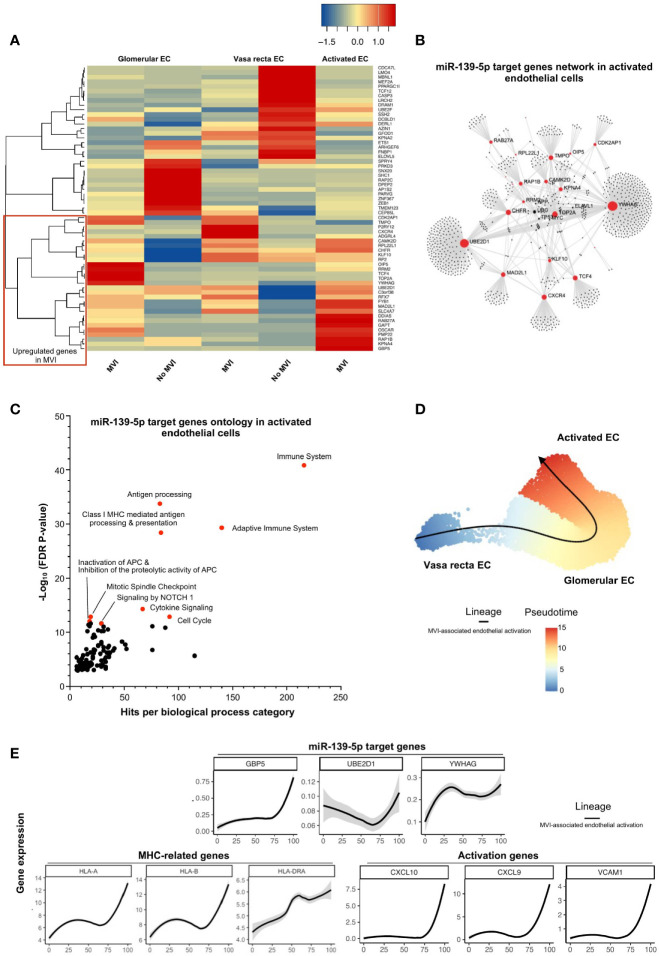
miR-139-5p decrease leads to activation of EC and antigen-presentation pathway during MVI. **(A)** Heatmap showing the expression of each individual miR-139-5p target genes in the different endothelial cells (EC) subsets and according to the MVI groups. Red rectangle indicates upregulated genes in MVI. **(B)** Gene network corresponding to those 31 upregulated genes in MVI built on Omicsnet. **(C)** Gene ontology and pathway enrichment analysis based on Omicsnet and Reactome databases. The name of the top 10 enriched pathways in term of adjusted P-value are shown (red dots). **(D, E)** Pseudotime analysis. **(D)** Pseudotime trajectories for EC based on Slingshot R package, showing one lineage (MVI-associated EC activation). **(E)** Profiling of genes along this trajectory to confirm their functional annotation.

MiR-139-5p was the only downregulated miRNA in our study in cases with MVI/ABMR (**Figure 2A**) and was found to be mainly originating from glomerular EC (**Figure 4A**). Sixty-one of its target genes were confirmed to be upregulated in bulk microarray from MVI+ cases (**Figure 3B**) and their expression was further investigated at single-cell resolution in the 3 previously identified EC subclusters (**Figures 4B, C**). As depicted in **Figure 5A**, the activated EC cluster was only detectable in MVI+ samples. Besides, half of the 61 genes (N=30) were consistently increased in sc-RNAseq MVI+ biopsies, regardless of the three EC subclusters. Among those, *GBP5* was the most increased gene. Next, gene ontology analysis using these 30 endothelial-derived transcripts identified *UBE2D1* and *YWHAG* as central players in the gene network (**Figure 5B**). More specifically, immune-related pathways and major histocompatibility complex (MHC)-mediated antigen processing and presentation pathways were enriched (**Figure 5C**). Pseudotime analysis was performed to better characterize gene expression changes in the course of EC activation. Across cells state trajectory (**Figures 5D, E**), *UBE2D1, YWHAG* and *GBP5* continuously increased during MVI associated endothelial activation. Very similar trajectories were found for MHC-related genes *HLA-A*, -*B* and -*DRA* but also for *bona-fide* endothelial activation and inflammation markers such as *VCAM1*, *CXCL9* and *CXCL10*, suggesting that all these genes are concomitantly expressed during endothelial activation. Altogether, these results suggest that miR-139-5p is decreased during MVI lesions and no longer repress the MHC antigen presentation pathway in activated EC.

### miR-222-3p Impairs Both Renal Physiological and Immune-Related Pathways in Epithelial Cells During MVI

**Figure 6:**
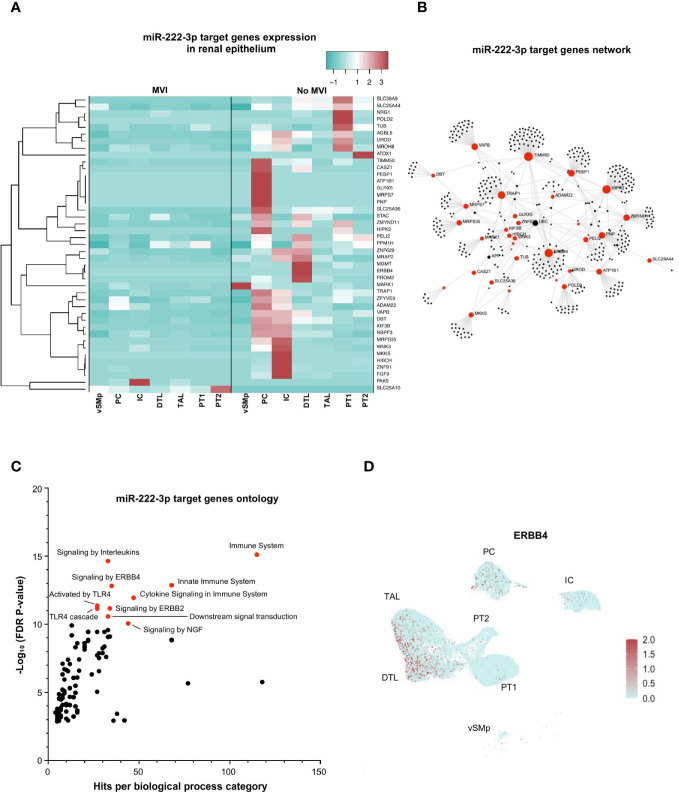
miR-222-3p impairs both renal physiological and immune-related pathways in epithelial cells during MVI. **(A)** Heatmap showing the expression of each individual miR-222-3p target genes in the different epithelial subsets and according to the MVI groups. **(B)** Gene network corresponding to the 43 downregulated genes in MVI genes built on Omicsnet. **(C)** Gene ontology and pathway enrichment analysis based on Omicsnet and Reactome databases. The name of the top 10 enriched pathways in term of adjusted P-value are shown (red dots). **(D)** UMAP showing a diffuse ERBB4 expression in all renal epithelial sc-RNAseq cell clusters. DTL, descending thick limb cells; IC, intercalated cells; PC, principal cells; PT, proximal tubular cells; TAL, thick ascending limb cells; UMAP, Uniform manifold approximation and projection; vSMp, vascular smooth muscle and pericytes.

Next, we focused on miR-222-3p, an upregulated miRNA in MVI samples with a renal epithelial cell origin and a more exclusive correlation to ABMR-related histological lesions (**Figures 2A, D**, **4A**). In bulk allograft tissue, the expression of 43 of its target genes was downregulated (**Figure 3B**), and further evaluated at a single-cell resolution in the different renal epithelial populations we previously identified (**Figures 4B, C**). A massive decrease of 41 of these 43 (95%) genes across all identified renal epithelial cell clusters suggests a gene-silencing profile in MVI (**Figure 6A**). Interestingly, 4 genes involved in renal physiological pathways (*ERBB4*, *ATP1B1*, *TIMM50* and *KIF3B*), but also immune-related genes (*TRAP1* and *PEBP1*) were identified as central genes in the network (**Figure 6B**). Gene ontology of the 43 genes revealed an enrichment in ErbB signaling pathways but also in immune-related pathways and cytokine response (**Figure 6C**) and *ERBB4* expression was further confirmed across all renal cells (**Figure 6D**). Altogether, these results suggest that during MVI, a high level of miR-222-3p in renal epithelial cells associates with strong repression of genes involved both in renal electrolyte homeostasis pathways and immune-related pathways in the epithelium.

### miR-150-5p Regulates NF-κB Activation in T Lymphocytes During MVI

**Figure 7:**
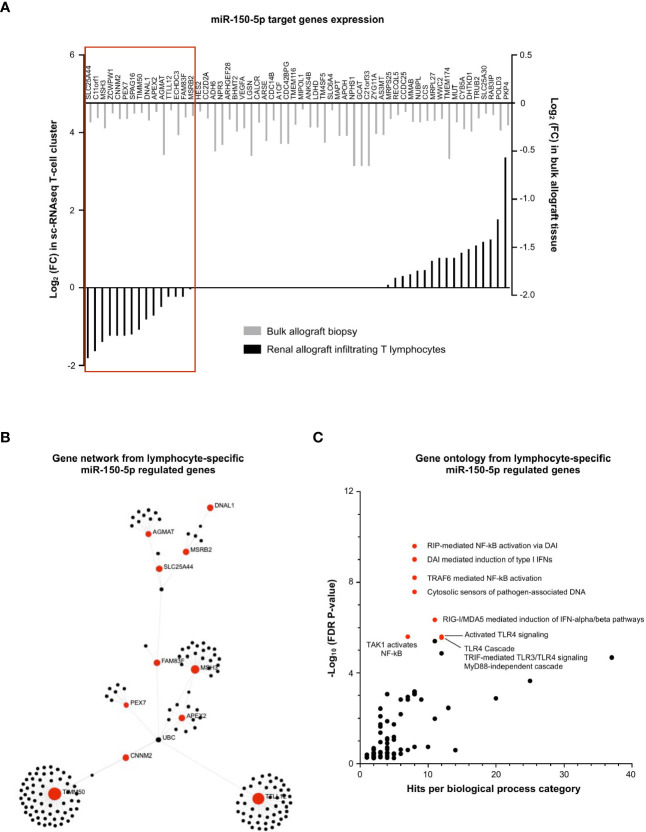
miR-150-5p regulates NF-κB activation in T lymphocytes during MVI. **(A)** Bar plots showing the differential expression of each individual miR-150-5p target genes between MVI + and MVI- samples, in the bulk transcriptomic analysis (light gray) or in the T lymphocytes cluster from sc-RNAseq analysis (black). Red frame indicates 15 genes, concordantly downregulated in MVI in bulk tissue and in the T lymphocyte cluster. **(B)** Gene network corresponding to the 15 downregulated genes in MVI built on Omicsnet. **(C)** Gene ontology and pathway enrichment analysis based on Omicsnet and Reactome databases. The name of the top 10 enriched pathways in term of adjusted P-value are shown (red dots).

Similarly, we investigated the regulatory role of (increased) miR-150-5p during MVI in the T-cell cluster, wherein it was most dominantly expressed (**Figure 4A**). We plotted the 58 decreased MVI-associated DEGs identified in bulk allograft biopsy samples (**Figure 3B**) and we confronted it to gene expression in the sc-RNAseq T-cell cluster (**Figure 7A**). The proportion of genes both targeted by miR150-5p and specifically underexpressed in T-cells during MVI was surprisingly low (15/58, 25.9%) suggesting that the global decrease of these genes *in vivo* was not exclusively due to the T lymphocyte compartment. Nevertheless, from this highly selected list of 15 genes and using OMICSnet platform, we constructed a gene network (**Figure 7B**) and performed gene ontology analysis (**Figure 7C**). We observed that miR-150-5p overexpression was associated with NF-κB regulation in T lymphocytes during MVI.

### miR-155-5p Regulates mRNA Splicing in APCs During MVI

**Figure 8:**
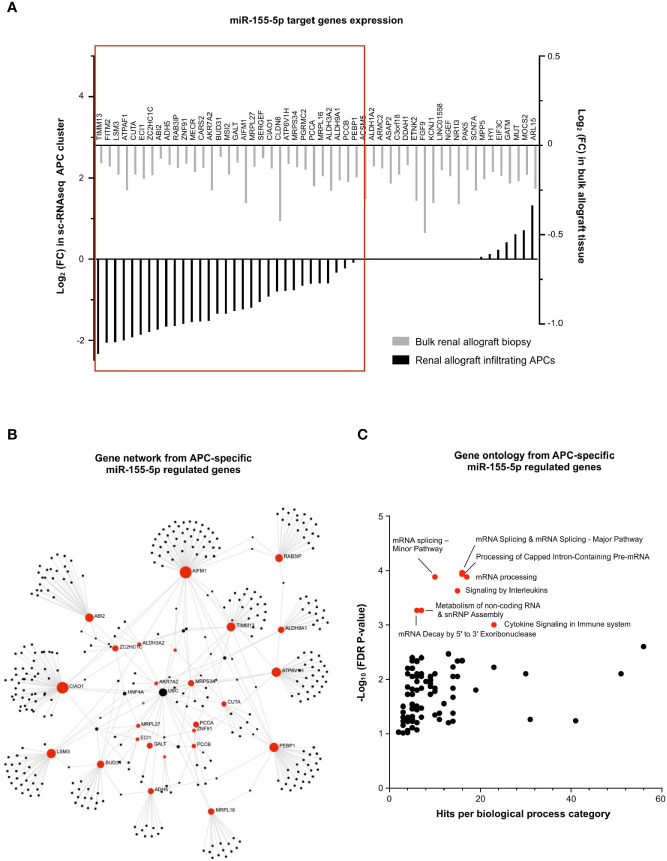
miR-155-5p regulates mRNA splicing in APCs during MVI. **(A)** Bar plots showing the differential expression of each individual miR-155-5p-target genes between MVI + and MVI- samples, in the bulk transcriptomic analysis (light gray) or in the APCs cluster from sc-RNAseq analysis (black). Red frame indicates 31 genes, concordantly downregulated in MVI in bulk tissue and in the APCs cluster. **(B)** Gene network corresponding to the 31 downregulated genes in MVI built on Omicsnet. **(C)** Gene ontology and pathway enrichment analysis based on Omicsnet and Reactome databases. The name of the top 10 enriched pathways in term of adjusted P-value are shown (red dots).

Finally, the exact same strategy was applied for the 52 miR-155-5p-target genes, underexpressed during MVI (**Figure 3B**). In contrast with miR-150-5p in T lymphocytes, we observed that almost two-third of the miR-155-5p targeted genes were consistently decreased in the APC sc-RNAseq cluster (31/52, 59.6%, **Figure 8A**). Gene network analysis revealed that *AIFM1* and *CIAO1* were central genes (**Figure 8B**). Moreover, gene ontology revealed a strong enrichment in pathways related to mRNA splicing (**Figure 8C**).

## Discussion

By its unique design, BIOMARGIN study is the typical framework for multi-omic data integration. Indeed, very robust pipelines were used in both discovery, selection and validation cohorts encompassing a large number of patients from different transplantation centers. In the three independent cohorts, we concomitantly observed and confirmed the peculiar expression profile of 6 miRNAs during MVI: miR-139-5p was decreased whereas miR-142-3p/150-5p/155-5p/222-3p and miR-223-3p were increased. Importantly, the level of each of these 6 miRNAs was correlated with MVI severity, suggesting a dose-dependent mechanism of regulation. We next used *in silico* platforms to investigate each miRNA cellular origin. Strikingly, two distinct groups of miRNAs were observed: a first derived from infiltrating immune cells and a second more specific to resident renal cells. Then, both miRNAs and mRNAs were measured in the same discovery set of biopsy samples, allowing an integrated analysis of *in vivo-*validated differentially expressed mRNAs/miRNAs associated with MVI. Finally, after describing mRNAs/miRNAs association at the bulk-tissue resolution, we confirmed mRNAs/miRNAs interplay at the single-cell resolution for four out of the 6 identified miRNAs.

Interestingly, miR-139-5p was the only miRNA negatively correlated with MVI lesions. It originated mainly from EC and has been reported to regulate MHC-related genes. Among its target genes, *UBE2D1* was highly upregulated during endothelial activation associated with MVI. UBE2D family is an E2 ubiquitin-conjugating enzyme family in the ubiquitin-proteasome system (29, 30) and UBeD1 was shown to enhance March-I ubiquitination, leading to the up-regulation of MHC class II proteins (31). In addition, *GBP5* was the most represented gene in activated EC during MVI, which is in line with our recent reports showing that GBP5 is highly upregulated in ABMR biopsies (17) but also in blood samples (32). Interestingly, *GBP5* expression was shown to be inducible by type II interferon in human uterine microvascular endothelial cells, together with *CXCL9* and *CXCL10* (33). The early role of these two proinflammatory chemokines in acute rejection processes is well documented and their level in urine is proposed to monitor the acute rejection risk in routine surveillance of kidney transplant recipient (34). Another miR-139-5p target is *YWHAG*. *YWHAG* codes for the protein family of HLA-F and has been described as differentially expressed during the active profibrotic process in diabetic nephropathy (35). The possible role of miR-139-5p in fibrosis, a final common pathway after inflammation, is all the more relevant that IFTA has recently been included in a composite score to predict kidney allograft survival after ABMR (36). Altogether, these results suggest that miR-139-5p no longer represses MCH antigen expression at the endothelium surface during MVI and could participate in chemoattraction and fibrosis processes, but these mechanisms remain to be experimentally confirmed.

The second renal-derived miRNA we identified is miR-222-3p, whose expression was found to be more specific of MVI lesions: it associated with g and ptc scores as well as with C4d deposition, but not with i or t lesions. However, it mainly originated from epithelial cells. After multi-omic integration at cell resolution, we determined that miR-222-3p mainly represses ErbB signaling which is crucial for fundamental cellular functions, such as proliferation, migration, growth, and differentiation (37). In human biology, physiological ErbB signaling is needed for renal electrolyte homeostasis and maintenance of kidney integrity (38). Our results also identified *ADAM22*, *NRG1*, *ZNF91* as ErbB-signaling related genes notably repressed in the epithelium during MVI. Other genes essential for kidney physiology such as *ATP1B1* and *KIF3B* were also repressed in renal epithelium by miR-222-3p during MVI. Previously, Baker et al. showed that ATP1B1 (β1 subunit of Na+/K+-ATPase) drives renal tubular reabsorption of sodium (39). In addition, Aguado-Fraile et al. have identified the Kinesin Family Member 3B (KIF3B) as involved in cell trafficking in rat proximal tubule cells. KIF3B appears as a key mediator of proximal epithelial tubule cell response to ischemia/reperfusion with potential application in renal ischemic damage management (40). We can thus speculate that miR-222-3p represses essential physiological pathways during MVI in the epithelium. Moreover, immune-related pathways are also regulated by miR-222-3p overexpression: important genes such as *TRAP1* and *PEBP1* were totally inhibited in samples with strong MVI. Interestingly, TRAP1 was shown to ameliorate renal tubulointerstitial fibrosis in mice with unilateral ureteral obstruction by protecting renal tubular epithelial cell mitochondria (41). Furthermore, PEBP1 provides anti-inflammatory effects through inhibition of both MAPK and NF-κB pathways under homeostatic/basal conditions (42). In renal epithelium, a study by Markó et al. elegantly showed that post-ischemic NF-κB activation aggravates tubular injury and exacerbates inflammation (43). In our hands, PEBP1 was completely repressed by miR-222-3p, suggesting that the NF-κB pathway is unleashed during MVI. Altogether, these results show that epithelial biology is disturbed during MVI and renal epithelial cells actively respond to MVI injuries while the involvement of this tissue in MVI is rarely addressed. These molecular findings might shed new light on acute tubular injury lesions, a well-established, yet long neglected ABMR criteria.

Besides, it has been shown that epithelial cells can secrete exosomal miR-222-3p and induce M2 phenotype in macrophages (44). In the meantime, Li et al. very recently reported that M2 macrophages overexpress miR-155 and deliver this miRNA in the microenvironment, also through secreting exosomes. In kidney transplantation, miR-155 was increased in various conditions (IFTA, acute rejection and TCMR) and in body fluid or tissues (45). Interestingly, miR-155 can promote epithelial-mesenchymal transition through targeting RASSF4 in epithelial cells (46). This miRNA-based crosstalk between macrophages and renal epithelial cells remains to be assessed in renal transplantation and more particularly in the context of MVI. In line with these findings, we observed that miR-155-5p was mainly expressed by APCs encompassing monocytes and macrophages. In this particular subset, miR-155-5p-target-genes ontology revealed enrichment in pre-mRNA splicing pathways. Interestingly, Janssen et al. reported that lung inflammation induced pre-mRNA splicing pathways in alveolar macrophages. Moreover, pre-mRNA splicing activation differed in tissue resident macrophages as compared to (blood-recruited) monocyte-derived macrophages. Changes in core metabolism in recruited macrophages were reported, with increased flux through glycolysis and decreased flux through the tricarboxylic acid cycle (TCA cycle, *i.e.* Krebs cycle) (47).

Concerning miR-150-5p, we found that its overexpression during MVI was associated with NF-κB pathway in the lymphocyte cluster encompassing both innate NKT cells and adaptive T cells. Interestingly, miR-150-5p is crucial to generate mature and functional NK cells (48) and miR-150-5p deficient CD8+ T cells were reported to be less cytotoxic (49). Beside, CD8 T cells can also secrete miR-150-5p in exosomes (50) that could in turn promote fibroblast activation in tubular epithelial cells and subsequent renal fibrosis as suggested by recent reports (51, 52).

Our study has some limitations. We focused our research on 6 miRNAs, consistently identified across our multistep design. However, we proceeded with a step-by-step miRNA selection, with only a fraction of all known miRNAs quantified in the validation cohort. The statistical pathway for miRNAs selection relied on BIOMARGIN’s initial design, suited to identify diagnostic biomarkers for multiple endpoints (ABMR, but also TCMR and IFTA). Hence, though significantly increased in the discovery and selection cohorts (**Figure 2A**), miR-146a was not quantified in the validation cohort. Thereby, miR-146a was *de facto* excluded from our selection process, though the literature suggests a role in ischemia/reperfusion in kidney transplantation (53) and in Treg-mediated control of alloimmune responses (54). Moreover, we cannot exclude that a more powerful sample-size would have yielded more miRNAs, such as miR-138 (down) and miR-511 (up) that were differentially expressed in ABMR cases of the selection cohort and showed a trend in the same direction in the discovery cohort.

Besides, we did not perform sc-RNAseq analysis on our own biopsy samples but used an online available sc-RNAseq dataset. At the time of study design and biopsy samples collection, sc-RNAseq technology was indeed not yet available, and the need for freshly-collected samples did not allow the retrospective use of frozen or paraffin-embed samples. Similarly, mRNA profiling was done using microarrays, the benchmark technology at the time which has now been outperformed by next-generation RNA-seq. The use of an online available sc-RNAseq dataset is limiting our ability of reporting the basic demographic characteristics of the patients. This missing information could raise concern regarding the representativity of population groups. Moreover, we did not control any technical aspect of the sequencing. Hence at the chosen resolution, our sc-RNAseq analysis did not detect any neutrophil or NK cells cluster in these samples. Therefore, our downstream analyses of miR-142-3p and miR-223-3p were limited to building their respective target gene network and ontology (**Supplementary Figure S2**) and did not allow any single-cell resolution exploration. However, the gene network and ontology of the 27 target genes of miR-223-3p revealed an enrichment of TCA cycle (**Supplementary Figure S2**) together with ERBB signaling. We can thus speculate that both miR-155-5p in monocytes and miR-223-3p in neutrophils play a role in metabolism and inflammatory response engaged by these cells during MVI. Besides, miR-142-3p has already been shown to be increased in kidney allograft rejection (9, 55, 56), but had so far not been specifically associated with ABMR. Another limitation of our study is that specific gene expression changes in the rare infiltrating cells might be masked out in bulk gene expression analysis. Further studies including more samples are thus required to allow proper investigation of all, even rare cell subtypes, but are currently limited by the relatively high cost of the technique.

In conclusion, we investigated here the molecular pathways associated with MVI, the main histological injury related to ABMR. The integrated omics approach used enabled us to unravel new pathways involved in MVI and suggests that tubular epithelial metabolism modifications occur as a consequence of ABMR, beyond the nearby endothelial compartment. Our study illustrates the great potential of multi-omics to unravel disease mechanisms and paves the way to new investigations to further elucidate the cross-talk between renal resident and allograft-infiltrating cell subsets.

## Data Availability Statement

The datasets presented in this study can be found in Gene Expression Omnibus (GEO) using the accession number GSE179772.

## Ethics Statement

The studies involving human participants were reviewed and approved by Ethics Committee of Ile-de-France XI (#13016). The patients/participants provided their written informed consent to participate in this study.

## Author Contributions

CT, BL, MN, and DA conceived and designed the present study. PM, DA, MN, and WG conceived the BIOMARGIN study and provided the allograft biopsy samples. LA collected the clinical data. CT and VS carried out the miRNA experiments, while JC conducted the transcriptomic profiling study. MR performed the histology reading of biopsies at Necker Hospital. CT, BL, and LM performed the statistical analyses and interpreted the data. JC and EL participated in the interpretation of the results. CT, BL, MN, and DA wrote the draft of the report. All the authors have read and approved the manuscript, and made important contributions to the manuscript.

## Funding

The BIOMARGIN study was funded by FP7 Health (Seventh Framework Programme of the European Commission), in the HEALTH.2012.1.4-1 theme of “innovative approaches to solid organ transplantation,” grant agreement no. 305499. This specific project was also supported by the ROCKET study, under the frame of ERACoSysMed-2, the ERA-Net for Systems Medicine in clinical research and medical practice, JTC2_29) and by funding from the Emmanuel Boussard Foundation.

## Conflict of Interest

The authors declare that the research was conducted in the absence of any commercial or financial relationships that could be construed as a potential conflict of interest.

## Publisher’s Note

All claims expressed in this article are solely those of the authors and do not necessarily represent those of their affiliated organizations, or those of the publisher, the editors and the reviewers. Any product that may be evaluated in this article, or claim that may be made by its manufacturer, is not guaranteed or endorsed by the publisher.

## Abbreviations

ABMR, antibody-mediated rejection; APC, antigen-presenting cell; DEG, differentially expressed gene; EC, endothelial cell; FDR, false-discovery rate; IFTA, interstitial fibrosis and tubular atrophia; KT, kidney transplantation; MHC, major histocompatibility complex; miRNA, micro ribonucleic acid; mRNA, messenger ribonucleic acid; MVI, microvascular inflammation; Sc-RNAseq, single-cell ribonucleic acid sequencing; TCMR, T-cell mediated rejection; UMAP, Uniform manifold approximation and projection.
