# Supplementary material for: Integrative Omics Analysis Unravels Microvascular Inflammation-Related Pathways in Kidney Allograft Biopsies
Source: Front Immunol. 2021 Nov 2;12:738795. doi: 10.3389/fimmu.2021.738795 (PMC8593247; doi:10.3389/fimmu.2021.738795)
Supplement: Supplementary file 1 [file DataSheet_1.docx]

Supplementary Material for

*Integrative omics analysis unravels microvascular inflammation-related pathways in kidney allograft biopsies*

**Table of contents**

SUPPLEMENTAL RESULTS Page 2

Supplemental Table 1 Page 2

Supplemental Table 2 Page 3

Supplemental Table 3 Page 4

Supplemental Table 4 Page 5

Supplemental Table 5 Page 6

Supplemental Table 6 Page 7

Supplemental Figure 1 Page 8

Supplemental Figure 2 Page 9

# Supplementary Results

## Supplementary Tables

**Table S1: Patient demographics in the discovery cohort.**

| **Variables** | **All samples**  ***N*=86** | **MVI-**  ***N*=59** | **MVI+**  ***N*=27** | ***P* value** |
| --- | --- | --- | --- | --- |
| Sex (men), *N (%)* | 55 (65.5%) | 39 (67.2%) | 16 (61.5%) | 0.6276 |
| Age at transplantation (yr), *mean±SD* | 50.5±13.9 | 51.3±13.5 | 48.9±14.9 | 0.5796 |
| Donor age (yr), *mean±SD* | 50.8±15.3 | 50.9±15.9 | 50.5±14.3 | 0.7206 |
| Living donor, *N (%)* | 17 (19.8%) | 14 (23.7%) | 3 (11.1%) | 0.2462 |
| Retransplantation, *N (%)* | 14 (16.3%) | 5 (8.5%) | 9 (33.3%) | 0.009 |
| CIT (h), *mean±SD ** | 15.4±5.6 | 14.5±5.1 | 17.2±6.1 | 0.1339 |
| Delayed graft function*, N (%)* | 14 (16.3%) | 7 (11.9%) | 7 (25.9%) | 0.1219 |
| **Cause of ESRD** |  |  |  |  |
| Glomerulonephritis, *N (%)* | 22 (25.6%) | 14 (23.7%) | 8 (29.6%) | 0.6003 |
| Diabetes, *N (%)* | 6 (7%) | 4 (6.8%) | 2 (7.4%) | 1 |
| Cystic/hereditary/congenital, *N (%)* | 27 (31.4%) | 19 (32.2%) | 8 (29.6%) | 1 |
| Secondary glomerulonephritis, *N (%)* | 4 (4.7%) | 2 (3.4%) | 2 (7.4%) | 0.5867 |
| Hypertension, *N (%)* | 3 (3.5%) | 2 (3.4%) | 1 (3.7%) | 1 |
| Interstitial nephritis, *N (%)* | 4 (4.7%) | 4 (6.8%) | 0 (0%) | 0.3039 |
| Miscellaneous conditions, *N (%)* | 9 (10.5%) | 8 (13.6%) | 1 (3.7%) | 0.2619 |
| Etiology uncertain, *N (%)* | 11 (12.8%) | 6 (10.2%) | 5 (18.5%) | 0.3099 |
| **Immunosuppressive protocol** |  |  |  |  |
| Induction therapy, *N (%)* | 65 (76.5%) | 42 (71.2%) | 23 (88.5%) | 0.1017 |
| Basiliximab, *N (%)* | 49 (57.6%) | 35 (59.3%) | 14 (53.8%) | 0.6431 |
| ATG, *N (%)* | 14 (16.5%) | 6 (10.2%) | 8 (30.8%) | 0.0269 |
| CNI: Cyclosporin, *N (%)* | 14 (16.3%) | 10 (16.9%) | 4 (14.8%) | 1 |
| Tacrolimus*, N (%)* | 72 (83.7%) | 49 (83.1%) | 23 (85.2%) | 1 |
| Mycophenolic acid, *N (%)* | 83 (96.5%) | 57 (96.6%) | 26 (96.3%) | 1 |
| mTOR inhibitors, *N (%)* | 1 (1.2%) | 1 (1.7%) | 0 (0%) | 1 |
| Steroids, *N (%)* | 86 (100%) | 59 (100%) | 27 (100%) | 1 |

**Table S2: Patient demographics in the selection cohort.**

| **Variables** | **All samples**  ***N*=99** | **No ABMR**  ***N*=71** | **ABMR**  ***N*=28** | ***P* value** |
| --- | --- | --- | --- | --- |
| Sex (men), *N (%)* | 63 (64.3%) | 46 (65.7%) | 17 (60.7%) | 0.6482 |
| Age at transplantation (yr), *mean±SD* | 48.6±15.5 | 50.2±14.3 | 44.6±17.9 | 0.1630 |
| Donor age (yr), *mean±SD* | 50.3±14.9 | 51.2±14 | 47.5±17.7 | 0.5494 |
| Living donor, *N (%)* | 19 (19.4%) | 13 (18.3%) | 6 (22.2%) | 0.7757 |
| Retransplantation, *N (%)* | 16 (16.2%) | 9 (12.7%) | 7 (25%) | 0.1428 |
| CIT (h), *mean±SD ** | 15.5±6.3 | 15.4±6.3 | 15.8±6.2 | 0.6241 |
| Delayed graft function*, N (%)* | 24 (25%) | 18 (25.7%) | 6 (23.1%) | 1 |
| **Cause of ESRD** |  |  |  |  |
| Glomerulonephritis, *N (%)* | 28 (28.3%) | 17 (23.9%) | 11 (39.3%) | 0.1431 |
| Diabetes, *N (%)* | 6 (6.1%) | 5 (7%) | 1 (3.6%) | 0.6722 |
| Cystic/hereditary/congenital, *N (%)* | 30 (30.3%) | 17 (23.9%) | 13 (46.4%) | 0.0504 |
| Secondary glomerulonephritis, *N (%)* | 4 (4%) | 3 (4.2%) | 1 (3.6%) | 1 |
| Hypertension, *N (%)* | 3 (3%) | 3 (4.2%) | 0 (0%) | 0.5564 |
| Interstitial nephritis, *N (%)* | 11 (11.1%) | 10 (14.1%) | 1 (3.6%) | 0.1725 |
| Miscellaneous conditions, *N (%)* | 2 (2%) | 1 (1.4%) | 1 (3.6%) | 0.4877 |
| Etiology uncertain, *N (%)* | 15 (15.2%) | 15 (21.1%) | 0 (0%) | 0.0051 |
| **Immunosuppressive protocol** |  |  |  |  |
| Induction therapy, *N (%)* | 64 (66.7%) | 43 (61.4%) | 21 (80.8%) | 0.0910 |
| Basiliximab, *N (%)* | 50 (52.1%) | 34 (48.6%) | 16 (61.5%) | 0.3582 |
| ATG, *N (%)* | 11 (11.5%) | 7 (10%) | 4 (15.4%) | 0.4820 |
| CNI: Cyclosporin, *N (%)* | 29 (29.6%) | 13 (18.6%) | 16 (57.1%) | <0.0001 |
| Tacrolimus*, N (%)* | 65 (66.3%) | 55 (78.6%) | 10 (35.7%) | <0.0001 |
| Mycophenolic acid, *N (%)* | 82 (83.7%) | 61 (87.1%) | 21 (75%) | 0.2241 |
| mTOR inhibitors, *N (%)* | 5 (5.1%) | 4 (5.7%) | 1 (3.6%) | 1 |
| Steroids, *N (%)* | 94 (95.9%) | 69 (98.6%) | 25 (89.3%) | 0.0692 |

**Table S3: Patient demographics in the validation cohort.**

| **Variables** | **All samples**  ***N*=298** | **No ABMR**  ***N=*269** | **ABMR**  ***N*=29** | ***P* value** |
| --- | --- | --- | --- | --- |
| Sex (men), *N (%)* | 182 (61.1%) | 170 (63.2%) | 12 (41.4%) | 0.0274 |
| Age at transplantation (yr), *mean±SD* | 51.5±14.9 | 52±14.5 | 47.2±18.2 | 0.2756 |
| Donor age (yr), *mean±SD* | 51±15.4 | 51.6±15.2 | 44.8±16.5 | 0.0474 |
| Living donor, *N (%)* | 61 (20.5%) | 57 (21.2%) | 4 (13.8%) | 0.4697 |
| Retransplantation, *N (%)* | 55 (18.5%) | 49 (18.2%) | 6 (20.7%) | 0.8009 |
| CIT (h), *mean±SD ** | 15.6±6.3 | 15.7±6.4 | 14.1±5.2 | 0.2144 |
| Delayed graft function*, N (%)* | 43 (14.6%) | 39 (14.6%) | 4 (14.3%) | 1 |
| **Cause of ESRD** |  |  |  |  |
| Glomerulonephritis, *N (%)* | 71 (23.8%) | 65 (24.2%) | 6 (20.7%) | 0.8202 |
| Diabetes, *N (%)* | 21 (7%) | 17 (6.3%) | 4 (13.8%) | 0.1342 |
| Cystic/hereditary/congenital, *N (%)* | 84 (28.2%) | 77 (28.6%) | 7 (24.1%) | 0.6711 |
| Secondary glomerulonephritis, *N (%)* | 14 (4.7%) | 13 (4.8%) | 1 (3.4%) | 1 |
| Hypertension, *N (%)* | 15 (5%) | 14 (5.2%) | 1 (3.4%) | 1 |
| Interstitial nephritis, *N (%)* | 30 (10.1%) | 27 (10%) | 3 (10.3%) | 1 |
| Miscellaneous conditions, *N (%)* | 23 (7.7%) | 20 (7.4%) | 3 (10.3%) | 0.4783 |
| Etiology uncertain, *N (%)* | 39 (13.1%) | 36 (13.4%) | 3 (10.3%) | 1 |
| **Immunosuppressive protocol** |  |  |  |  |
| Induction therapy, *N (%)* | 227 (76.2%) | 205 (76.2%) | 22 (75.9%) | 1 |
| Basiliximab, *N (%)* | 177 (59.4%) | 159 (59.1%) | 18 (62.1%) | 0.8437 |
| ATG, *N (%)* | 44 (14.8%) | 41 (15.2%) | 3 (10.3%) | 0.5925 |
| CNI: Cyclosporin, *N (%)* | 22 (7.4%) | 18 (6.7%) | 4 (13.8%) | 0.2493 |
| Tacrolimus*, N (%)* | 272 (91.3%) | 248 (92.2%) | 24 (82.8%) | 0.1544 |
| Mycophenolic acid, *N (%)* | 262 (87.9%) | 238 (88.5%) | 24 (82.8%) | 0.3689 |
| mTOR inhibitors, *N (%)* | 34 (11.4%) | 31 (11.5%) | 3 (10.3%) | 1 |
| Steroids, *N (%)* | 295 (99%) | 267 (99.3%) | 28 (96.6%) | 0.2653 |

**Table S1-3 abbreviations:** ABMR, antibody-mediated rejection; ATG, anti-thymocyte globulin; CIT, cold ischemia time; CNI, calcineurin inhibitor; ESRD, end-stage renal disease; MVI, microvascular inflammation; SD, standard deviation; Yr, year; mTOR, mechanistic target of rapamycin. * for deceased donor.

**Table S4: Histological characteristics in the discovery cohort.**

| **Elementary lesions** | **All samples**  ***N*=86** | **MVI-**  ***N*=59** | **MVI+**  ***N*=27** | ***P* value** |
| --- | --- | --- | --- | --- |
| Sample quality: adequate/limit. *N (%)* | 81 (94.2%) | 57 (96.6%) | 24 (88.9%) | 0.1758 |
| Number of glomeruli. *mean*±*SD* | 21.6±10.9 | 23.6±11.5 | 16.6±7.7 | 0.0049 |
| g score >0. *N (%)* | 21 (24.7%) | 0 (0%) | 21 (77.8%) | <0.0001 |
| g score. *mean*±*SD* | 0.5±0.9 | 0±0 | 1.5±1.1 | <0.0001 |
| ptc score >0. *N (%)* | 24 (28.6%) | 0 (0%) | 24 (88.9%) | <0.0001 |
| ptc score. *mean*±*SD* | 0.5±0.9 | 0±0 | 1.6±0.9 | <0.0001 |
| i score >0. *N (%)* | 17 (20.2%) | 9 (15.8%) | 8 (29.6%) | 0.1564 |
| i score. *mean*±*SD* | 0.4±0.8 | 0.3±0.8 | 0.6±1 | 0.1399 |
| t score >0. *N (%)* | 22 (26.2%) | 9 (15.8%) | 13 (48.1%) | 0.0030 |
| t score. *mean*±*SD* | 0.4±0.8 | 0.2±0.6 | 0.7±0.9 | 0.0014 |
| v score >0. *N (%)* | 3 (3.8%) | 0 (0%) | 3 (11.1%) | 0.0356 |
| v score. *mean*±*SD* | 0.1±0.4 | 0±0 | 0.2±0.7 | 0.0146 |
| cg score >0. *N (%)* | 9 (10.6%) | 1 (1.7%) | 8 (29.6%) | <0.0001 |
| cg score. *mean*±*SD* | 0.2±0.7 | 0±0.1 | 0.7±1.2 | <0.0001 |
| ci score >0. *N (%)* | 48 (56.5%) | 30 (51.7%) | 18 (66.7%) | 0.2433 |
| ci score. *mean*±*SD* | 1±1 | 0.9±1.1 | 1±0.9 | 0.4933 |
| ct score >0. *N (%)* | 54 (63.5%) | 34 (58.6%) | 20 (74.1%) | 0.2276 |
| ct score. *mean*±*SD* | 1±1 | 1±1.1 | 1.1±0.9 | 0.4780 |
| cv score >0. *N (%)* | 41 (50.6%) | 26 (48.1%) | 15 (55.6%) | 0.6388 |
| cv score. *mean*±*SD* | 0.9±1 | 0.7±0.9 | 1.1±1.2 | 0.1585 |
| ah score >0. *N (%)* | 42 (49.4%) | 29 (50%) | 13 (48.1%) | 1 |
| ah score. *mean*±*SD* | 0.8±1 | 0.8±0.9 | 1±1.2 | 0.6598 |
| ti score >0. *N (%)* | 35 (41.2%) | 22 (37.9%) | 13 (48.1%) | 0.4785 |
| ti score. *mean*±*SD* | 0.7±1.1 | 0.7±1.1 | 0.9±1.1 | 0.4028 |
| c4d score >0. *N (%)* | 7 (9%) | 1 (1.8%) | 6 (27.3%) | 0.0016 |
| c4d score. *mean*±*SD* | 0.2±0.8 | 0.1±0.4 | 0.7±1.3 | <0.0001 |

**Table S5: Histological characteristics in the selection cohort.**

| **Elementary lesions** | **All samples**  ***N*=99** | **No ABMR**  ***N*=71** | **ABMR**  ***N*=28** | ***P* value** |
| --- | --- | --- | --- | --- |
| Sample quality: adequate/limit. *N (%)* | 83 (83.8%) | 62 (87.3%) | 21 (75%) | 0.1428 |
| Number of glomeruli. *mean*±*SD* | 19.2±8.7 | 19.5±8.6 | 18±9.3 | 0.3052 |
| g score >0. *N (%)* | 15 (15.5%) | 3 (4.3%) | 12 (44.4%) | <0.0001 |
| g score. *mean*±*SD* | 0.3±0.7 | 0±0.2 | 0.8±1 | <0.0001 |
| ptc score >0. *N (%)* | 19 (19.2%) | 4 (5.6%) | 15 (53.6%) | <0.0001 |
| ptc score. *mean*±*SD* | 0.3±0.6 | 0.1±0.4 | 0.8±0.8 | <0.0001 |
| i score >0. *N (%)* | 9 (9.1%) | 8 (11.3%) | 1 (3.6%) | 0.4386 |
| i score. *mean*±*SD* | 0.2±0.6 | 0.2±0.6 | 0.1±0.6 | 0.2553 |
| t score >0. *N (%)* | 23 (23.2%) | 12 (16.9%) | 11 (39.3%) | 0.0323 |
| t score. *mean*±*SD* | 0.4±0.8 | 0.3±0.8 | 0.5±0.8 | 0.0333 |
| v score >0. *N (%)* | 6 (6.7%) | 2 (3%) | 4 (16.7%) | 0.0413 |
| v score. *mean*±*SD* | 0.1±0.5 | 0.1±0.4 | 0.2±0.5 | 0.0282 |
| cg score >0. *N (%)* | 13 (13.4%) | 0 (0%) | 13 (48.1%) | <0.0001 |
| cg score. *mean*±*SD* | 0.3±0.8 | 0±0 | 1.1±1.3 | <0.0001 |
| ci score >0. *N (%)* | 52 (52.5%) | 31 (43.7%) | 21 (75%) | 0.0069 |
| ci score. *mean*±*SD* | 1±1.1 | 0.9±1.2 | 1.3±1 | 0.0287 |
| ct score >0. *N (%)* | 58 (58.6%) | 36 (50.7%) | 22 (78.6%) | 0.0131 |
| ct score. *mean*±*SD* | 1.1±1.1 | 0.9±1.1 | 1.4±1 | 0.0393 |
| cv score >0. *N (%)* | 57 (63.3%) | 39 (59.1%) | 18 (75%) | 0.2186 |
| cv score. *mean*±*SD* | 1.2±1.1 | 1±1 | 1.5±1.2 | 0.0607 |
| ah score >0. *N (%)* | 55 (55.6%) | 35 (49.3%) | 20 (71.4%) | 0.0715 |
| ah score. *mean*±*SD* | 1±1.1 | 0.8±1 | 1.5±1.2 | 0.0065 |
| ti score >0. *N (%)* | 14 (14.1%) | 5 (7%) | 9 (32.1%) | 0.0027 |
| ti score. *mean*±*SD* | 0.2±0.7 | 0.1±0.6 | 0.5±0.8 | 0.0019 |
| c4d score >0. *N (%)* | 4 (4.4%) | 1 (1.5%) | 3 (11.5%) | 0.0688 |
| c4d score. *mean*±*SD* | 0.1±0.6 | 0±0.4 | 0.3±0.9 | 0.0389 |

**Table S6: Histological characteristics in the validation cohort.**

| **Elementary lesions** | **All samples**  ***N*=298** | **No ABMR**  ***N=*269** | **ABMR**  ***N*=29** | ***P* value** |
| --- | --- | --- | --- | --- |
| Sample quality: adequate/limit. *N (%)* | 296 (99.7%) | 268 (99.6%) | 28 (100%) | 1 |
| Number of glomeruli. *mean*±*SD* | 24.1±12.7 | 24.1±12.8 | 25±11.1 | 0.4995 |
| g score >0. *N (%)* | 37 (12.4%) | 9 (3.3%) | 28 (96.6%) | <0.0001 |
| g score. *mean*±*SD* | 0.2±0.6 | 0±0.2 | 1.6±0.9 | <0.0001 |
| ptc score >0. *N (%)* | 40 (13.4%) | 14 (5.2%) | 26 (89.7%) | <0.0001 |
| ptc score. *mean*±*SD* | 0.2±0.7 | 0.1±0.4 | 1.8±0.9 | <0.0001 |
| i score >0. *N (%)* | 12 (4%) | 8 (3%) | 4 (13.8%) | 0.0208 |
| i score. *mean*±*SD* | 0.1±0.3 | 0±0.2 | 0.2±0.7 | 0.0044 |
| t score >0. *N (%)* | 28 (9.4%) | 23 (8.6%) | 5 (17.2%) | 0.1695 |
| t score. *mean*±*SD* | 0.1±0.5 | 0.1±0.5 | 0.2±0.5 | 0.1439 |
| v score >0. *N (%)* | 5 (1.7%) | 1 (0.4%) | 4 (13.8%) | <0.0001 |
| v score. *mean*±*SD* | 0±0.2 | 0±0.1 | 0.2±0.6 | <0.0001 |
| cg score >0. *N (%)* | 17 (5.7%) | 4 (1.5%) | 13 (44.8%) | <0.0001 |
| cg score. *mean*±*SD* | 0.1±0.4 | 0±0.1 | 0.8±1.1 | <0.0001 |
| ci score >0. *N (%)* | 168 (56.6%) | 152 (56.5%) | 16 (57.1%) | 1 |
| ci score. *mean*±*SD* | 1±1.1 | 1±1 | 1.2±1.3 | 0.4683 |
| ct score >0. *N (%)* | 166 (55.9%) | 150 (55.8%) | 16 (57.1%) | 1 |
| ct score. *mean*±*SD* | 1±1.1 | 1±1 | 1.2±1.3 | 0.4151 |
| cv score >0. *N (%)* | 188 (65.5%) | 167 (64.7%) | 21 (72.4%) | 0.5373 |
| cv score. *mean*±*SD* | 1.2±1.1 | 1.1±1 | 1.4±1.2 | 0.2259 |
| ah score >0. *N (%)* | 151 (51.2%) | 133 (50%) | 18 (62.1%) | 0.2445 |
| ah score. *mean*±*SD* | 0.8±1 | 0.8±0.9 | 1.2±1.2 | 0.0522 |
| ti score >0. *N (%)* | 37 (12.5%) | 32 (11.9%) | 5 (18.5%) | 0.3566 |
| ti score. *mean*±*SD* | 0.2±0.5 | 0.2±0.5 | 0.4±0.9 | 0.2680 |
| c4d score >0. *N (%)* | 75 (25.3%) | 64 (24%) | 11 (37.9%) | 0.1163 |
| c4d score. *mean*±*SD* | 0.4±0.8 | 0.3±0.7 | 0.8±1.2 | 0.0413 |

**Table S4-6 abbreviations:** ABMR, antibody-mediated rejection; ah, arteriolar hyalinosis; cg, allograft glomerulopathy; ci, interstitial fibrosis; ct, tubular atrophy; cv, chronic vascular changes; C4d, degradation product of the activated complement factor C4; g, glomerulitis; i, interstitial infiltrate; MVI, microvascular inflammation; ptc, peritubular capillaritis; SD, standard deviation; t, tubulitis; ti, total inflammation; v, vasculitis. Patients with mixed rejection were included in the ABMR group: selection cohort *N*=4, validation cohort *N*=1

## Supplementary Figures

**Figure S1: Single-cell-RNAseq identification markers used for cell cluster annotation**

Dot plot showing selected cell marker gene expression (dots color scale) and within-cluster detection rate (dots size).

T-cells and NKT cells were identified as CD3E and CD8A expressing cells, as well as using IL7R expression, a commonly accepted surrogate marker for CD4. Antigen-presenting cells (APCs) mainly encompassed monocytes and HLA-DRA and MS4A7 expressing macrophages, but also B cells expressing CD19. We also identified EC expressing PECAM1, which were further refined in three EC clusters: vasa recta EC (ECvr) expressing CLDN5 and FLT1, glomerular EC (ECg) expressing PLVAP and EMCN and activated EC (ECa) expressing high level of VCAM1. In addition, nephron epithelial cells were evident such as principal (PC) and intercalated cells (IC) expressing TMEM213 and SLC4A1 respectively, and also 2 clusters of proximal tubular cells (PT) both expressing CUBN and LRP2 but different expression of PDZK1IP1, as previously reported (23). Other cells such as vascular smooth muscle and pericytes (vSMp) expressing ACTA2 and MYH11 and Loop of Henle thick ascending limb cells (TAL) expressing UMOD and descending thick limb cells (DTL) expressing ITGB6 were identified.

Abbreviations: APCs, antigen-presenting cells; DTL, descending thick limb cells; ECa, activated endothelial cells; ECg, glomerular endothelial cells; ECvr, vasa recta endothelial cells; IC, intercalated cells; PC, principal cells; PT, proximal tubular cells; TAL, thick ascending limb cells; vSMp, vascular smooth muscle and pericytes.


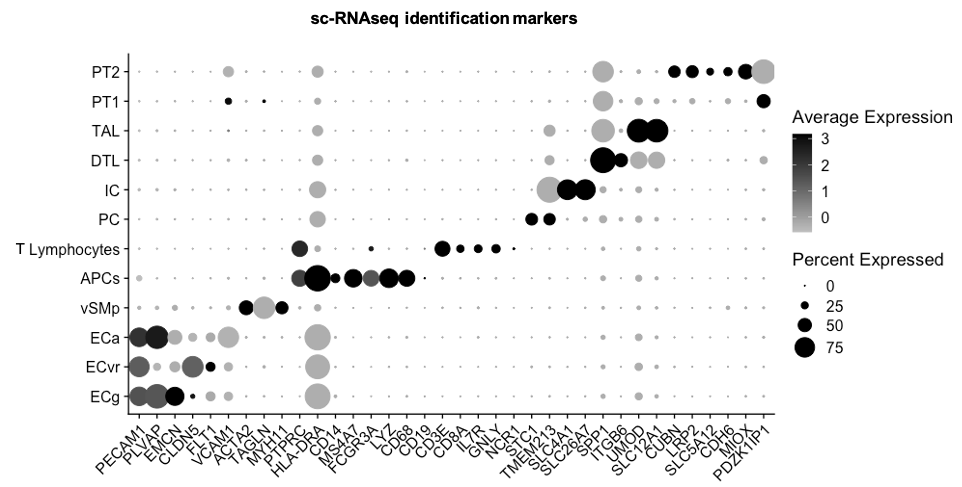


**Figure S2: miR-223-3p and miR-142-3p target genes analysis**

**(A, C)**. Gene networks for miR-223-3p **(A)** and miR-142-3p **(C)** *in vivo* decreased gene targets from the transcriptomic analysis. **(B, D)**. Gene ontology and pathway enrichment analysis for miR-223-3p **(B)** and miR-142-3p **(D)** based on Omicsnet and Reactome databases. The name of the top 10 enriched pathways in term of adjusted P-value are shown (red dots).

**
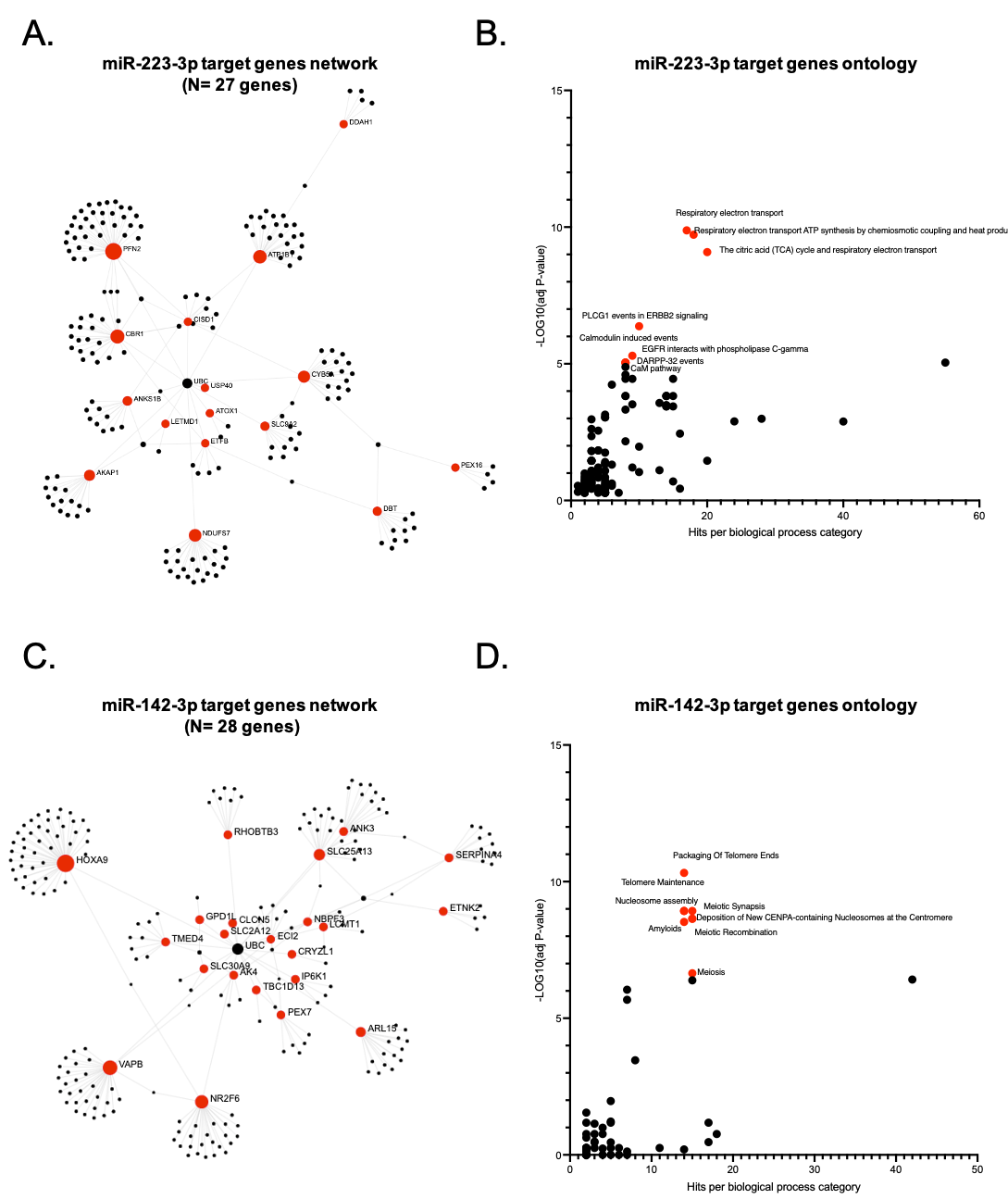
**
